# The profile of Day Program participants at the South Australian Statewide Eating Disorder Service

**DOI:** 10.1186/2050-2974-3-S1-P16

**Published:** 2015-11-23

**Authors:** Eva Vall, Angeline Kuek, Randall Long, Corree Guerin, Emma Altman, Tracey Wade

**Affiliations:** 1 School of Psychology, Flinders University, Bedford Park, Australia; 2 Statewide Eating Disorder Service, Southern Adelaide Local Health Network, Adelaide, Australia

## 

The Weight Disorder Unit at Flinders Medical Centre was established in 1977 and in June 2014, the service was expanded under a rebranded name, the South Australian Statewide Eating Disorder Service (SEDS). Now, besides its inpatient unit, SEDS also has outpatient services and an eating disorder Day Program. This is the first Day Program in South Australia and besides providing nutritional support, the Day Program also focuses on helping participants to challenge disordered eating behaviours and thought patterns through group-based therapeutic interventions. To be admitted into the SEDS Day Program, clients need to be at least 15 years of age, have a Body Mass Index (BMI) of 15 and above and be medically stable. In order to gain a better understanding of the profile of clients that get admitted to day programs and the effectiveness of the Day Program, we plan to present the clinical profile of the participants and information collected from the SEDS Day Program. Information presented will include sociodemographic and medical information, information collected from clients' psychological assessments pre, post and 3 months follow-up after completion of the Day Program, and the retention rate of the Day Program participants.

